# Potential Modulatory Microbiome Therapies for Prevention or Treatment of Inflammatory Bowel Diseases

**DOI:** 10.3390/ph14060506

**Published:** 2021-05-26

**Authors:** Daan V. Bunt, Adriaan J. Minnaard, Sahar El Aidy

**Affiliations:** 1 Host-Microbe Interaction, Groningen Biomolecular Sciences and Biotechnology Institute (GBB), University of Groningen, 9747 AG Groningen, The Netherlands; d.v.bunt@rug.nl; 2 Stratingh Institute for Chemistry, University of Groningen, Nijenborgh 7, 9747 AG Groningen, The Netherlands; a.j.minnaard@rug.nl

**Keywords:** microbial metabolites, gut, inflammation

## Abstract

A disturbed interaction between the gut microbiota and the mucosal immune system plays a pivotal role in the development of inflammatory bowel disease (IBD). Various compounds that are produced by the gut microbiota, from its metabolism of diverse dietary sources, have been found to possess anti-inflammatory and anti-oxidative properties in in vitro and in vivo models relevant to IBD. These gut microbiota-derived metabolites may have similar, or more potent gut homeostasis-promoting effects compared to the widely-studied short-chain fatty acids (SCFAs). Available data suggest that mainly members of the Firmicutes are responsible for producing metabolites with the aforementioned effects, a phylum that is generally underrepresented in the microbiota of IBD patients. Further efforts aiming at characterizing such metabolites and examining their properties may help to develop novel modulatory microbiome therapies to treat or prevent IBD.

## 1. Introduction

Inflammatory bowel disease (IBD) is an idiopathic disease affecting the gastrointestinal (GI) tract and can be divided into two main subcategories: Crohn’s disease (CD) and ulcerative colitis (UC). Both CD and UC lead to poor quality of life and psychological distress for patients, and produce significant pressure on healthcare systems by their relatively high morbidity. Genetic and environmental factors are known to increase the risk of IBD and may predispose certain individuals or populations to developing the disease. Prevalence of IBD has always been relatively high in Europe and North America, but is now also on the rise in industrializing countries in Asia, Africa, and South America [1].

Despite the lack of full understanding of the pathophysiology of IBD, the majority of available reports suggest a dysregulation between the intestinal microbiota and the host immune system (i.e., loss of immune tolerance) to be one of the underlying causes. The innate immune system in the intestinal mucosa responds to the microbiota and/or antigens by promoting inflammation, which recruits the adaptive immune system and leads to a more severe and long-lasting inflammatory state, as well as deterioration of the intestinal barrier integrity. The latter leads to translocation of microbiota and/or antigens into the mucosa, further exacerbating the mucosal inflammatory response, thereby creating a vicious circle [2,3].

Currently used pharmacological interventions are aimed at combatting the characteristic flareups of intestinal inflammation. The most effective drugs are corticosteroids and tumor necrosis factor (TNF) inhibitors. However, the former cannot be used for extended periods of time due to serious side effects (e.g., Cushing’s syndrome), and the latter has a significant amount of primary and secondary non-responders, along with serious side effects [4,5,6].

Fecal microbiota transplant (FMT) is another, experimental, form of IBD treatment. A recent meta-analysis found that 54% of IBD patients showed a clinical response to FMT, and 37% demonstrated clinical remission, while 29% suffered from adverse events [7]. Generally, the adverse events following FMT are mild and subside within 24 h, but more serious events, such as IBD flareups, infections, colectomy, pancreatitis, and death are also reported, although less frequently [8].

Despite the promising remission rates of this IBD treatment, which is still in its infancy, the main motive against FMT is that the treatment is considered to be a black box. The outcome and safety of the treatment is influenced by a myriad of factors (e.g., host genotype, specific type of microbiota imbalance, type and stage of IBD, route of administration, and factors related to the FMT donor), which remain obscure [8,9].

Considering the pivotal role of the gut microbiota in IBD, and that, ultimately, a major part of the communication between the gut microbiota and the host is based on chemical signaling, this review aims to examine gut microbial metabolites known to have anti-IBD effects. In order to positively implicate the role of microbial metabolism, only compounds proven to be produced by the gut microbiota have been taken into consideration. Furthermore, the metabolites discussed in this review originate from parental compounds found in common dietary sources (e.g., vegetables, fruits, and herbs), and have either been shown to improve colitis symptoms in vivo, affect signaling pathways involved in the pathophysiology of IBD in vitro, or both. The relevant data are summarized in Table 1.

Due to intrinsic differences in the interindividual dietary and microbiota compositions, especially the disturbed microbiota of IBD patients, such metabolites may not be produced universally. Identifying these metabolites can help to overcome such intrinsic differences, and, ideally, helps making gut health less dependent on changes in the microbiota composition.

## 2. Indoles

**Figure 1:**
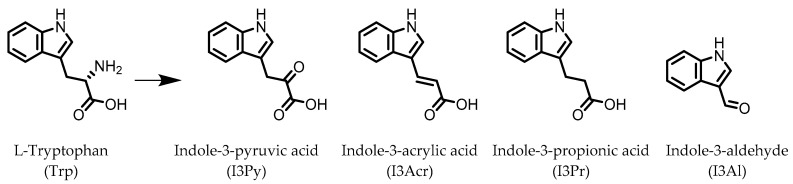
Structures of L-tryptophan and several indole metabolites produced by the gut microbiota.

Indole derivatives (Figure 1) are mainly produced by Lactobacilli, Clostridia, Peptostreptococci, Bifidobacteria, and Bacteroides (Table 1), as metabolites of the amino acid tryptophan (Trp) [95]. Gut microbial Trp metabolites are often found to be agonists of the aryl hydrocarbon receptor (AHR), of which lower levels are observed in IBD patients, compared to healthy subjects [96]. IBD symptoms and pro-inflammatory cytokine levels were found to be greater in AHR knockouts in murine models of dextran sodium sulfate (DSS)-induced colitis [97]. Other AHR ligands are known to reduce colitis symptoms [96,98].

AHR activation by the gut microbial Trp metabolite indole-3-aldehyde (I3Al) was shown to stimulate mucosal lymphocytes to secrete interleukin 22 (IL-22), an anti-inflammatory cytokine known to play an important role in protecting mice from developing IBD [99]. Increased IL-22 secretion causes signal transducer and activator of transcription 3 (STAT3) phosphorylation, which ultimately leads to faster proliferation of intestinal epithelial cells (IECs), contributing to the recovery of damaged intestinal mucosa following DSS-induced colitis [10].

Indole-3-propionic acid (I3Pr) also activates the AHR receptor, which induced IL-10 receptor expression in cultured IECs. Oral administration of I3Pr was shown to improve DSS-induced murine colitis symptoms, which was attributed to increased signaling of the anti-inflammatory cytokine IL-10, due to higher expression of IL-10 receptors [13].

Additionally, I3Pr was found to act as a ligand for the pregnane X receptor (PXR) in vivo, and led to lower TNF-α levels together with higher levels of mRNA coding for tight junction proteins, thus contributing to intestinal integrity. With the help of knockout experiments, it was determined that activation of PXR modulates Toll-like receptor 4 (TLR4) signaling, which is known to activate nuclear factor κB (NF-κB), a pro-inflammatory transcription factor. Accordingly, oral administration of I3Pr could activate PXR in the colon, which prevents lipopolysaccharide (LPS)-induced inflammation via modulation of TLR4, thereby preserving the intestinal integrity [14].

Administration of indole-3-pyruvic acid (I3Py) to mice with CD4^+^ T cell-induced colitis led to an increase in the amount of IL-10-producing T cells, while the number of Th1 cells in the mucosa was decreased, resulting in a reduction in colitis symptoms [19].

In a co-culture of murine-derived colonic spheroids and murine bone marrow-derived macrophages (BMDMs), indole-3-acrylic acid (I3Acr) promoted IL-10 secretion while suppressing TNF-α production upon stimulation with LPS, via activation of AHR. This stimulated the expression of the mucin protein coding gene, *Muc2*, which may help to protect the intestinal epithelium. When human peripheral blood mononuclear cells (PBMCs) were treated with I3Acr, a reduction in IL-1β and IL-6 was observed, upon LPS stimulation. Moreover, not only was AHR activation reproduced in the human cell line, activation of the anti-inflammatory Nrf2–ARE pathway was observed. Using these human PBMCs in the co-culture, I3Acr treatment promoted important anti-inflammatory and anti-oxidant effects, by upregulating Nrf2- and AHR-pathway target genes and genes related to the biosynthesis glutathione (GSH), an important anti-oxidant that protects cells from oxidative stress [20].

## 3. Urolithins

**Figure 2:**
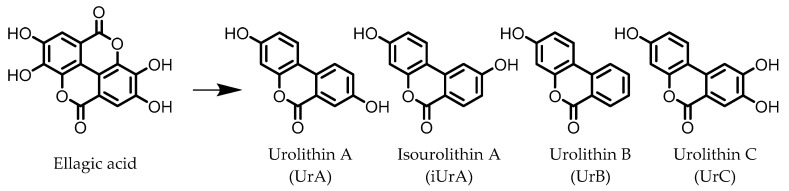
Structures of ellagic acid and several urolithins produced by the gut microbiota.

Urolithins are gut microbial metabolites of ellagic acid, a hydrolysis product of ellagitannins (Figure 2). Both ellagic acid and ellagitannins are naturally found in various fruits, nuts, and seeds (e.g., pomegranate, raspberry, strawberry, almond, and walnut) [100]. Several members of the Actinobacteria (Table 1) have been found to metabolize ellagic acid into particular urolithins, which differ by the number and the positions of hydroxyl groups. For example, *Gordonibacter urolithinfaciens* and *Gordonibacter pamelaeae* are able to produce urolithin C (UrC), but are not capable of further dehydroxylation [29,30]. Urolithin A (UrA) and urolithin B (UrB) are produced by *Bifidobacterium pseudocatenulatum*, whereas isourolithin A (iUrA) is produced by *Ellagibacter isourolithinifaciens* [21,27,28].

A comparison between the effects of pomegranate extract (PE) and UrA on DSS-induced colitis in rats showed that both were able to decrease levels of the pro-inflammatory mediators nitric oxide (NO) and prostaglandin E_2_ (PGE_2_) in colonic mucosa, by downregulating the enzymes responsible for their production: inducible nitric oxide synthase (iNOS), cyclooxygenase 2 (COX-2), and prostaglandin E synthase (PTGES). However, only in the case of UrA administration was the colonic architecture protected. Additionally, UrA was able to significantly downregulate the pro-inflammatory cytokines IL-1β and IL-4, and cluster of differentiation 40 (CD40), a receptor protein involved in immune and inflammatory signaling pathways [22].

It was also observed that less UrA was produced from PE in colitic rats compared to healthy rats, suggesting that UrA production from gut microbiota, which might be absent in inflammation, plays a protective role against colitis. During colitis, UrA itself had to be administered in order to benefit from the anti-inflammatory effects. Another protective effect of UrA might be via an observed increase in the abundance of Lactobacilli, Bifidobacteria, and Clostridia taxa, which have been shown to prevent inflammation in IECs in response to pathogenic Enterobacteria [101]. Moreover, an increase in *E. coli*, observed after DSS treatment, was found to be lower in the rats that received UrA [22].

Several in vitro studies have been performed in an attempt to reveal a more detailed mechanism explaining the anti-inflammatory actions of UrA. The production of pro-inflammatory mediators was strongly reduced by UrA in LPS-stimulated RAW264 macrophages. UrA was found to inhibit the phosphorylation of protein kinase B (Akt) and c-Jun, effectively suppressing the pro-inflammatory PI3-K/Akt/NF-κB and JNK/AP-1 signaling pathways. This meant the downstream production of pro-inflammatory mediators (TNF-α, IL-6, and NO) was also suppressed. Notably, UrA appeared to also inhibit NADPH oxidase (NOX), which is largely responsible for production of reactive oxygen species (ROS) in activated macrophages, presenting another possible mechanism for inhibiting the activation of the pro-inflammatory transcription factors NF-κB and AP-1 [23].

iUrA, UrB, and UrC also display anti-inflammatory effects in LPS-stimulated RAW264.7 macrophages, although the effects are inferior to UrA. The urolithins were shown to decrease the DNA-binding activity of the NF-κB p50 subunit, as well as the nuclear translocation of the p65 subunit, resulting in lower levels of TNF-α, IL-1β, IL-6, iNOS, and NO [24,25]. Additionally, UrA has been shown to promote anti-inflammatory effects in human macrophages and neutrophils, which was attributed to an observed induction of extracellular signal-regulated kinase 1 and 2 (ERK1/2) phosphorylation [25].

Besides the anti-inflammatory properties and modulation of the microbiota, UrA can also improve gut health by enhancing the intestinal barrier function. UrA was shown to activate AHR and Nrf2, which leads to the upregulation of the tight junction proteins claudin 4, occludin, and zonula occludens-1 (ZO-1). Treatment with UrA decreased gut permeability in mice with 2,4,6-trinitrobenzene sulfonic acid (TNBS)-induced colitis, and reduced both local and systemic inflammation. When UrA was administered prior to TNBS-administration, the development of colitis was prevented. Finally, chronic and acute DSS-induced colitis were ameliorated by UrA treatment [26].

## 4. Enterodiol (ED) and Enterolactone (EL)

**Figure 3:**
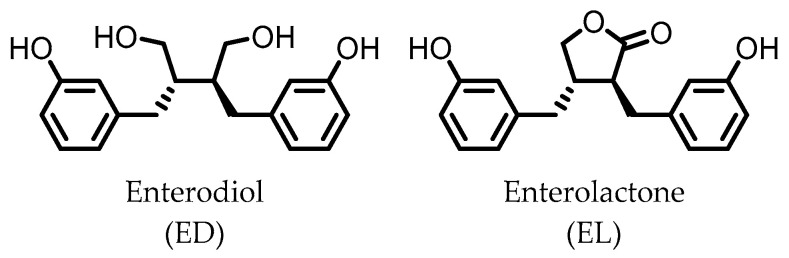
Structures of enterodiol and enterolactone.

Enterodiol (ED) and its oxidation product enterolactone (EL) (Figure 3) are formed by the intestinal microbiota upon lignan consumption. Lignans are polyphenolic compounds found in seeds, nuts, and vegetables. Production of ED and EL from naturally occurring lignans is dependent on the combined metabolic activities of different species [31]. However, several members of the Actinobacteria and Firmicutes phyla have been implicated in catalyzing the final step towards ED and/or EL (Table 1) [31,32,33,34,35,36,37,38].

Both ED and EL are able to pass the intestinal barrier and have been found to suppress the release of TNF-α from THP-1 human monocytes upon LPS stimulation. This observation was attributed to an inhibitory effect of ED and EL on the degradation of IκB (inhibitor of NF-κB), leading to lower NF-κB activity. EL was found to be more active than ED [39].

Additionally, EL was shown to reduce oxidative stress damage in LPS-stimulated RAW264.7 cells, and in a co-culture of Caco2/RAW264.7 cells, EL treatment maintained barrier integrity. Experiments on HCT-8 human colonocytes, with TNF-α and interferon γ (INF-γ)-induced inflammation, showed that EL significantly increased expression of peroxisome proliferator-activated receptor γ (PPAR-γ) and tight junction protein ZO-1, suggesting a mechanism for the observed maintenance of barrier integrity [40].

## 5. Flavonoids

**Figure 4:**
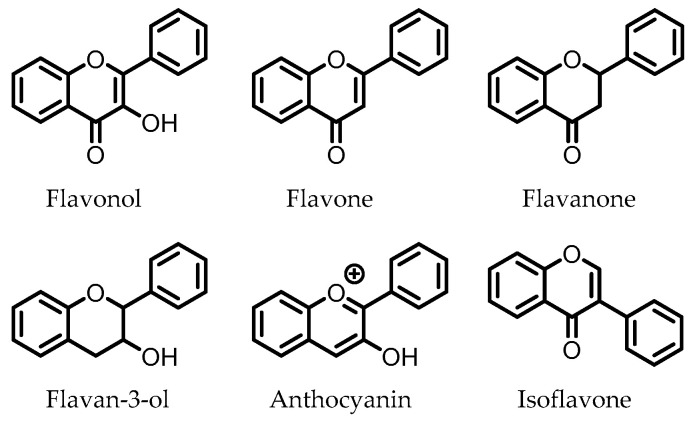
Core structures of flavonoid subclasses. The phenyl rings may be functionalized at different positions (i.e., -OH, -OMe).

Flavonoids are polyphenolic compounds that consist of the subclasses flavonols, flavanones, flavones, flavan-3-ols, anthocyanins, and isoflavones, of which the core structures are depicted in Figure 4. These compounds are present in various foods and drinks, and are commonly found as glycosides. Flavonoid glucosides are often hydrolyzed in the small intestine, where, subsequently, the flavonoid aglycone can be absorbed. Flavonoid glycosides that contain other sugars than glucose do not undergo hydrolysis in the small intestine and can reach the colon intact. There, the intestinal microbiota are able to cleave the glycosidic bonds to release the respective flavonoid aglycones [102].

Flavonoids have been consistently found to produce anti-inflammatory effects throughout various tissues, including the GI tract, and have been shown to ameliorate experimental colitis models [103,104,105,106,107,108]. It has also been repeatedly shown that flavonoid aglycones have stronger anti-inflammatory activities than their glycosides [109]. This suggests that the anti-IBD effects observed for flavonoid glycosides are dependent on microbial deglycosylation in the colon to release the flavonoid aglycones.

**Figure 5:**
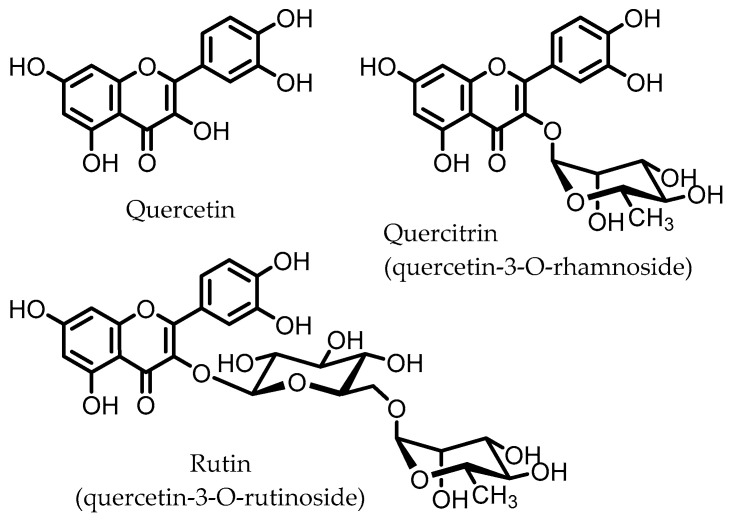
Structures of quercetin, quercitrin, and rutin.

Quercitrin and rutin are glycosides of quercetin (Figure 5), one of the most common flavonols, which can be found in various vegetables and fruits. These quercetin glycosides are not enzymatically hydrolyzed or absorbed in the small intestine and can reach the colon, where diverse microbes (Table 1) catalyze the deglycosylation to produce the aglycone quercetin [110].

Quercetin was found to inhibit the NF-κB pathway in macrophages in vitro, inhibiting the expression of IL-1β, TNF-α, and NOS. These effects were not observed for the glycoside quercitrin. However, in vivo studies on rats with DSS-induced colitis showed that quercitrin, not quercetin, facilitated the recovery of inflamed mucosa. These results indicate that the active compound is quercetin, but that its glycoside quercitrin has to be orally administered for quercetin to be released in the colon via hydrolysis by the intestinal microbiota. This conclusion was supported by microbial fermentation experiments, showing that the intestinal microbiota are able to hydrolyze quercitrin to produce quercetin [41,42].

Similar results have been obtained for rutin; oral administration to mice with the CD4^+^ CD62L^+^ T cell-induced colitis model, which is closer to the human IBD compared to chemically induced colitis, led to improvement of colitis symptoms and a marked reduction in colonic levels of myeloperoxidase (MPO), an ROS-producing enzyme. Expression of pro-inflammatory genes (IFN-γ, TNF-α, IL-1β, CXCL1, and S100A8) was significantly reduced, as well as pro-inflammatory cytokine plasma levels. In vitro studies on splenocytes and murine T cells showed that the aglycone quercetin displayed a concentration-dependent inhibition of pro-inflammatory cytokine release, whereas rutin itself did not show such effects [45]. Hence, it is likely that the aglycone quercetin is responsible for the anti-colitic effects that are observed in vivo, and that this is due to the microbiota-catalyzed hydrolysis of rutin in the colon.

Other studies have shown that rutin, but not quercetin, was able to improve DSS-induced colitis by attenuating the expression of pro-inflammatory cytokines IL-1β and IL-6, and that rutin may prevent depletion of colonic GSH, reducing the damage arising from oxidative stress, and thereby promote colonic healing in TNBS-induced rat colitis [46,47].

Unfortunately, possible involvement of the gut microbiota is often not taken into consideration for in vivo studies in which the compound in question is administered orally. Thus, such studies are commonly performed using flavonoid aglycones, which may not reach the colon due to absorption in the small intestine. This means the gut microbiota cannot be implicated in observed anti-IBD effects of orally administered flavonoid aglycones, although the gut microbiota-mediated deglycosylations of the respective glycosides have often been described [54,55]. Without comparison of the glycoside and aglycone of flavonoids, the role of the gut microbial deglycosylation in promoting anti-IBD effects remains speculative.

Despite these discrepancies, the gut microbiota may play another role in the observed anti-IBD effects of orally administered flavonoids. Besides deglycosylation, various members of the gut microbiota have been found to catabolize flavonoid aglycones into smaller phenolic acids. In some cases, these phenolic acids appeared to have stronger anti-inflammatory effects than the parental compounds. For example, cyanidin-3-glucoside (C3G) is known to be hydrolyzed by the gut microbiota to the aglycone cyanidin. Both C3G and cyanidin have been shown to improve chemically induced colitis [111]. However, it has been found that protocatechuic acid (PCA), a further gut microbial metabolite of cyanidin, has a stronger anti-colitic effect than C3G, suggesting that the effects are dependent on the production of PCA from C3G and/or cyanidin [56,57].

Interestingly, it has recently been shown that fecal samples of healthy human subjects produce significantly higher levels of the phenolic acid metabolites 3-hydroxyphenylpropionic acid (3HPP), 3,4-dihydroxyphenylacetic acid (DHPA), and 3,4-dihydroxyphenyl-γ-valeric lactone (DHPVL) upon fermentation of polyphenols, compared to subjects with moderate to severe UC [112]. These data suggest that phenolic acids may be involved in gut homeostasis.

## 6. Dihydroxylated Phenolic Acids

**Figure 6:**
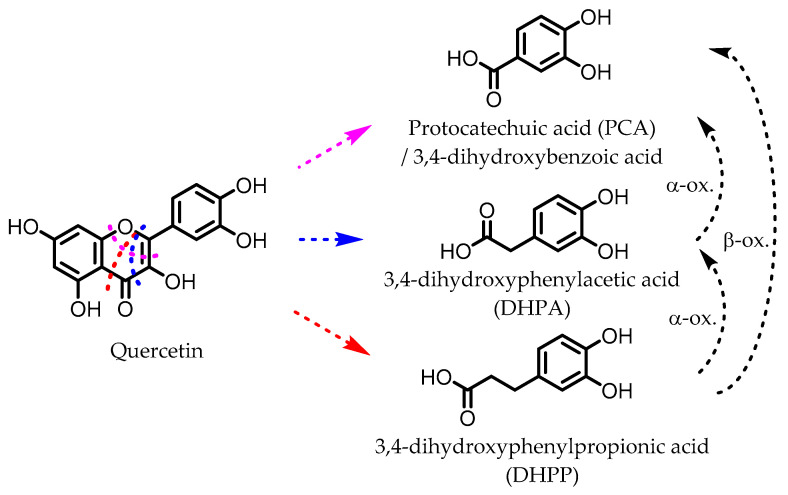
Example of proposed metabolic pathways that give phenolic acids from a flavonol (here: quercetin). The colored dotted lines indicate the possible different ring fissions, while the black dotted arrows indicate α- and β-oxidation.

PCA, DHPA, and DHPP are gut microbial catabolites of flavonols, flavones, flavan-3-ols, and anthocyanins. Several members of the Firmicutes (Table 1) have been found to be able to catalyze the ring fission of those flavonoids that is required to produce these metabolites [54,55,58,59,60,61,62,63]. It is unclear whether the different carbon chain lengths are the result of distinct ring fissions of the flavonoid, or that PCA and DHPA are produced from DHPP via α- and/or β-oxidation, as shown in Figure 6 [58,60,113].

Oral administration of PCA improved symptoms of DSS-induced colitis in rats and prevented the increase in the pro-inflammatory cytokines IL-1β, IL-6, and TNF-α that was seen in controls. Moreover, MPO activity and the concentrations of important markers for oxidative stress, NO, H_2_O_2_, and malondialdehyde (MDA), were reduced, while GSH levels were increased [64].

Similar results were obtained for mice with TNBS-induced colitis, and an investigation into the mechanism of the anti-inflammatory and anti-oxidant effects led to the possible explanation that PCA modulates SphK/S1P signaling, which serves as an important pathway for activation of STAT3 and NF-κB [65].

PCA was also found to increase the Firmicutes/Bacteroidetes ratio in LPS-challenged piglets. An increase in expression of tight junction proteins ZO-1 and claudin 1 was also observed in the intestinal mucosa, which may have been related to the accompanied decrease in pro-inflammatory cytokines IL-2 and TNF-α [66].

DHPA and DHPP decreased PGE_2_ production in IL-1β-stimulated CCD-18 colon fibroblasts. DHPP treatment improved DSS-induced colitis symptoms in rats and lowered the expression of IL-1β, IL-8, and TNF-α. Furthermore, MDA levels and oxidative damage to DNA were reduced in distal colon mucosa [67].

DHPA and DHPP significantly inhibited the release of TNF-α, IL-1β, and IL-6 in LPS-stimulated PBMCs and were also found to induce glutathione S-transferase theta-2 (GSTT2) expression while decreasing that of COX-2 in LT87 human colon cells [68,69].

## 7. Gallic Acid (GA)/3,4,5-trihydroxybenzoic Acid

**Figure 7:**
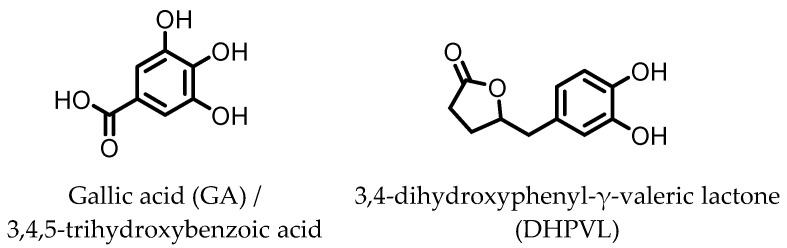
Structures of gallic acid and 3,4-dihydroxyphenyl-γ-valeric lactone.

GA (Figure 7) has been found to be produced from anthocyanins in similar fashion to the earlier mentioned dihydroxylated phenolic acids. GA was shown to ameliorate various chemically induced murine colitis models [70,71,72,73,74,75]. A decrease in the expression of the pro-inflammatory cytokines IL-1β, IL-6, IL-12, IL-17, IL-21, IL-23, TNF-α, IFN-γ, and transforming growth factor β (TGF-β) was observed, while an increased expression of the anti-inflammatory cytokines IL-4 and IL-10 was measured. Additionally, the activity and/or expression of COX-2, iNOS, and MPO was decreased, while those of superoxide dismutase (SOD), catalase (CAT), glutathione peroxidase (GPx), and glutathione reductase (GR) were increased. These effects are likely due to the observed decrease in pSTAT3 expression and NF-κB activity. The latter may be explained by an increase in IκBα expression and decrease in p65-NF-κB expression [70]. Moreover, the level of anti-oxidant transcription factor Nrf2 was significantly higher with GA administration [71].

## 8. 3,4-Dihydroxyphenyl-γ-valeric Lactone

One of the major gut microbial metabolites of flavan-3-ols and proanthocyanins is 3,4-dihydroxyphenyl-γ-valeric lactone (DHPVL) [114,115]. DHPVL (Figure 7) showed a dose-dependent decrease in NO production and iNOS expression in RAW264.7 macrophages, whereas the metabolic precursor catechin (flavan-3-ol) did not. The compound was also found to accumulate in macrophages and human monocytes in vitro. This was attributed to facilitated transport proteins, as the uptake was shown to decrease in the presence of the influx inhibitor phloretin [78]. Furthermore, a dose-dependent inhibition of NF-κB activity has been observed in TNF-α-stimulated HepG2 human liver cells [79].

Via chemical synthesis, both enantiomers of DHPVL were obtained separately and their anti-inflammatory mechanism on IEC-6 rat small intestine epithelial cells was investigated. The phosphorylation and degradation of IκBα in LPS-stimulated IEC-6 cells was prevented by (*S*)-DHPVL more than by (*R*)-DHPVL. Moreover, (*S*)-DHPVL showed a dose-dependent inhibition, and suggests that (*S*)-DHPVL is able to reduce NF-κB activation by inhibiting IκBα degradation, preventing LPS-induced inflammation [80].

## 9. Berberine

**Figure 8:**
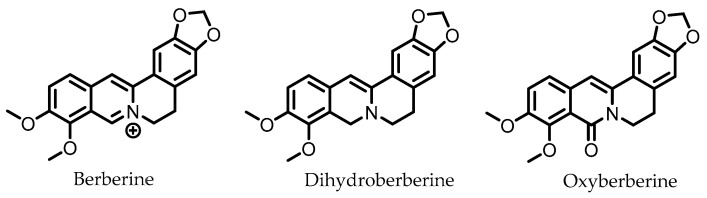
Structures of berberine and its gut microbial metabolites dihydroberberine and oxyberberine.

Berberine (Figure 8) is an alkaloid found in several herbs (e.g., *Coptis chinensis*) used for traditional Chinese medicine. Pharmacokinetic studies on rats have shown that the largest portion of orally ingested berberine ends up the feces. In humans, berberine also exhibits poor oral bioavailability, suggesting it mainly persists in the GI lumen until excreted in the feces, consistent with rat studies [116]. The gut microbiota is known to metabolize berberine into various compounds. The metabolite dihydroberberine has received the most attention, since it has lost the positive charge of the parental compound berberine, and is absorbed in the gut much more efficiently. Once absorbed, it is re-oxidized to berberine [116].

In vitro studies have shown that berberine is able to improve intestinal barrier integrity in Caco-2 cells, and prevent the redistribution of tight junction proteins in Caco-2 cells when treated with the pro-inflammatory cytokines TNF-α and IFN-γ, protecting the intestinal barrier. The underlying mechanism is believed to involve the suppression of the NF-κB pathway [81,82]. In a mouse model of endotoxemia (LPS injection), berberine was indeed able to prevent the redistribution of tight junction proteins in IECs by reducing NF-κB activity [83].

Various other mechanisms, through which berberine may reduce colitis symptoms, have been proposed. Berberine suppresses IFN-γ- and IL-17A-releasing LPCD4^+^ T cells by activation of AMP-activated protein kinase (AMPK) in vitro and in vivo. The latter led to reduced colonic inflammation in mice with T cell-induced chronic colitis [84]. Moreover, berberine was found to decrease the expression of the IL-6-related pro-inflammatory cytokine Oncostatin M (OSM) and its receptor OSMR, which are known to be involved in activating the JAK–STAT signaling pathway, an important early signaling pathway that leads to inflammation in response to extracellular pro-inflammatory cytokines [85]. This study also showed a marked improvement of colitis symptoms upon berberine administration in mice treated with DSS, along with a rectification of tight junction protein and *Muc2* expression, protecting the intestinal barrier integrity. Additionally, berberine treatment prior to acetic acid-induced colitis in rats was shown to downregulate p38 mitogen-activated protein kinase (MAPK) and upregulate Nrf2 expression, which may have been responsible for the observed downstream effects. These include reductions in colitis symptoms, pro-inflammatory markers TNF-α, IL-1β, IL-6, MPO, and PGE_2_, and levels of NO and MDA, whereas TGF-β expression, GSH levels, and enzyme activities of SOD, CAT, GPx, and GR, were increased [86].

Oxyberberine, a recently identified gut microbial metabolite of berberine, was found to have superior anti-colitic effects with respect to berberine. Oral administration of oxyberberine to mice with DSS-induced colitis significantly reduced colitis symptoms, inflammation, and disruption of the intestinal barrier. Oxyberberine targets the TLR4/MyD88/NF-κB pathway on multiple levels: expressions of TLR4 and MyD88, a protein involved in signal relaying, were reduced, phosphorylation of IκBα was inhibited, and reduced levels of p65 NF-κB were observed in the nucleus [88].

## 10. Ginsenosides

Ginsenosides are triterpenoid glycosides that are found in plants of the *Panax* genus, which have been widely used in traditional medicine. These compounds can be subdivided into glycosides of protopanaxadiol (PPD) and protopanaxatriol (PPT). Due to the polarity of the glycosyl groups, the oral bioavailability of naturally occurring ginsenosides is generally low. The gut microbiota is known to remove these sugars to produce secondary glycosides and, ultimately, aglycones, which are more readily absorbed by the host. Because of the different core structures and varying patterns of glycosylation, there is a relatively large variety of possible gut microbial ginsenoside metabolites [89,90].

**Figure 9:**
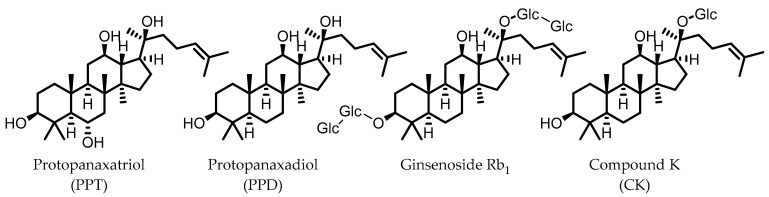
Structures of protopanaxatriol, protopanaxadiol, ginsenoside Rb1, and compound K. (Glc = glucose).

Compound K (CK), a glycoside of PPD and only one glucose unit (Figure 9), is considered the most important gut microbial ginsenoside metabolite in terms of bioactivities, which include anti-colitic effects. Mice that were fed American ginseng showed a significant improvement in DSS-induced colitis symptoms, and had reduced levels of the pro-inflammatory cytokines IL-1β and IL-6. CK was identified as a major metabolite of the used ginseng that was specifically produced by the intestinal microbiota, as mice treated with a broad-spectrum antibiotic did not have CK in their stool. CK was shown to inhibit IL-8 secretion from LPS-stimulated HT-29 cells, even at low concentrations, whereas ginsenoside Rb_1_, the major constituent of American ginseng, did not [91]. These results strongly indicate that the gut microbial conversion of ginsenosides into CK is responsible for the observed anti-colitic effects.

Additionally, in separate studies, CK was shown to target the NF-κB pathway in LPS-stimulated murine macrophage models, leading to reduced expressions of TNF-α, IL-1β, and IL-6. The study that used murine peritoneal macrophages showed an additional increase in expression of the anti-inflammatory cytokine IL-10 and reported that the potency of CK was superior to that of ginsenoside Rb_1_. Moreover, these studies showed that CK helped to reduce colitis symptoms in mice, induced by DSS or TNBS [92,93].

CK was also identified as a modulator of PXR/NF-κB signaling, to which the anti-colitic effects on DSS-treated mice were attributed. The authors propose that the attenuation of intestinal inflammation by CK restored the expression of PXR, but also found that CK appears to enhance the interaction between PXR and the p65 subunit of NF-κB, inhibiting NF-κB activity. Via this mechanism, CK is thought to stimulate PXR/NF-κB signaling only in inflamed colon cells, and helps to restore it to normal levels. As CK is not an agonist of PXR, there is no danger of PXR overactivation in a non-inflammatory state [94].

Besides CK, other gut microbial metabolites of ginsenosides have been found to possess anti-inflammatory and anti-IBD effects via various mechanisms [90,117].

## 11. Conclusions and Outlook

IBD is a multifactorial disease that is not fully understood. Genetics, environmental factors, and lifestyle play different roles in distinct cases. Undoubtedly, the interplay between the gut microbiota and the host immune system has a pivotal role in the disease, which is characterized by periodic flareups of intestinal inflammation.

Different dietary and herbal compounds appear to be metabolized by the gut microbiota into compounds with various anti-inflammatory and anti-oxidant properties as detected in in vitro as well as in in vivo animal models (Table 1). The downstream effects include improved intestinal integrity, reduced levels of pro-inflammatory cytokines and oxidants, and improvement of colitis symptoms.

The gut microbial fermentation products of dietary fibers, short-chain fatty acids (SCFAs), have been well studied and are regarded as important anti-inflammatory compounds that are crucial for maintaining gut homeostasis [118]. The studies discussed in this review provide a glimpse of various other gut microbial metabolites from dietary sources that may have similar, or possibly more potent effects with respect to SCFAs, based on the in vitro and in vivo studies performed (Table 1). It is noteworthy that Firmicutes is the predominant phylum responsible for production of these metabolites, as a reduction in Firmicutes is consistently observed in patients with IBD compared to healthy subjects, although it is unclear whether this reduction is a cause or result of IBD [119].

The relatively limited number of studies focusing on the anti-inflammatory and/or anti-IBD effects of specific gut microbiota-derived metabolites presented here, shows that there are more compounds involved in gut homeostasis than is generally assumed, and that these compounds can originate from diverse dietary sources. The studies also show the complicated nature of the interplay between host and gut microbiota, in the context of IBD and its intervention or prevention. Further efforts into characterizing gut microbial metabolites of dietary compounds, and experiments on models that mimic human IBD in vivo, may ultimately lead to novel IBD modulatory microbiome therapies or postbiotics. Such therapies would not be dependent on the composition of the microbiota, but rather on their metabolic products, and could be a better alternative to existing treatments, such as FMT.

## Figures and Tables

**Table 1:** Overview of metabolites, bacterial species currently known to produce these metabolites, and experimental models used to assess anti-IBD effects.

Microbial Metabolite	Parental Compound	Phylum	Species	Experimental Model	Ref.
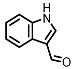 Indole-3-aldehyde (I3Al)	Tryptophan	Firmicutes	*Lactobacillus reuteri* *Lactobacillus murinus*	in vitro, in vivo	[10,11,12]
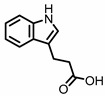 Indole-3-propionic acid (I3Pr)	Tryptophan	Firmicutes	*Peptostreptococcus russellii* *Peptostreptococcus anaerobius* *Peptostreptococcus asaccharolyticus* *Clostridium sporogenes* *Clostridium botulinum* *Clostridium caloritolerans* *Clostridium paraputrificum* *Clostridium cadaveris*	in vitro, in vivo	[13,14,15,16,17,18]
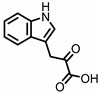 Indole-3-pyruvic acid (I3Py)	Tryptophan	Firmicutes	*Clostridium sporogenes*	in vitro, in vivo	[15,19]
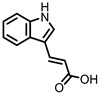 Indole-3-acrylic acid (I3Acr)	Tryptophan	Firmicutes	*Peptostreptococcus russellii* *Peptostreptococcus anaerobius* *Clostridium sporogenes*	in vitro	[15,20]
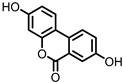 Urolithin A (UrA)	Ellagic acid	Actinobacteria	*Bifidobacterium pseudocatenulatum*	in vitro, in vivo	[21,22,23,24,25,26]
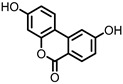 Isouroithin A (iUrA)	Ellagic acid	Actinobacteria	*Ellagibacter isourolithinifaciens*	in vitro	[25,27,28]
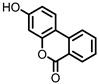 Urolithin B (UrB)	Ellagic acid	Actinobacteria	*Bifidobacterium pseudocatenulatum*	in vitro	[21,24,25]
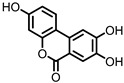 Urolithin C (UrC)	Ellagic acid	Actinobacteria	*Gordonibacter urolithinfaciens* *Gordonibacter pamelaeae*	in vitro	[24,29,30]
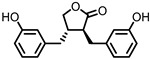 Enterolactone (EL) 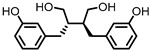 Enterodiol (ED)	Lignans	Firmicutes	*Lactobacillus gasseri* *Lactobacillus salivarius* *Clostridium scindens* *Lactonifactor longoviformis* *Peptostreptococcus productus*	in vitro	[31,32,33,34,35,36,37,38,39,40]
		Actinobacteria	*Bifidobacterium bifidum* *Bifidobacterium catenulatum* *Bifidobacterium pseudolongum* *Bifidobacterium adolescentis* *Eggerthella lenta*		
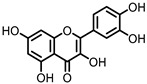 Quercetin	Quercitrin	Fusobacteria	*Fusobacterium K-60*	in vitro, in vivo	[41,42,43,44]
	Rutin	Firmicutes	*Enterococcus avium* *Lactobacillus acidophilus* *Lactobacillus plantarum* *Lachnoclostridium* spp. *Eisenbergiella* spp. *Blautia* sp.	in vitro, in vivo	[45,46,47,48,49,50,51,52,53]
		Actinobacteria	*Bifidobacterium dentium*		
		Bacteroidetes	*Bacteroides uniformis* *Bacteroides ovatus* *Parabacteroides distasonis*		
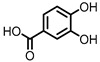 Protocatechuic acid (PCA)/3,4-dihydroxybenzoic acid 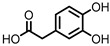 3,4-dihydroxyphenylacetic acid (DHPA) 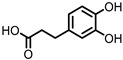 3,4-dihydroxyphenylpropionic acid (DHPP)	Flavonols Flavan-3-ols Flavones Anthocyanins	Firmicutes	*Eubacterium oxidoreducens* *Eubacterium ramulus* *Enterococcus casseliflavus* *Flavonifractor plautii* *Catenibacillus scindens* *Butyrivibrio* spp.	in vitro, in vivo	[54,55,56,57,58,59,60,61,62,63,64,65,66,67,68,69]
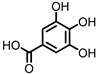 Gallic acid (GA)/3,4,5-trihydroxybenzoic acid	Anthocyanins	Firmicutes	*Lactobacillus plantarum* *Lactobacillus casei*	in vitro, in vivo	[70,71,72,73,74,75,76,77]
		Actinobacteria	*Bifidobacterium lactis*		
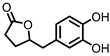 3,4-dihydroxyphenyl-γ-valeric lactone (DHPVL)	Flavan-3-ols Proanthocyanins	Firmicutes	*Lactobacillus plantarum* *Clostridium coccoides* *Flavonifractor plautii*	in vitro	[54,55,58,59,60,63,78,79,80]
		Actinobacteria	*Eggerthella lenta* *Eggerthella* sp.		
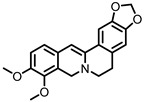 Dihydroberberine	Berberine	Firmicutes	*Enterococcus faecium* *Enterococcus faecalis* *Staphylococcus aureus* *Staphylococcus epidermis*	in vitro ^a^, in vivo	[81,82,83,84,85,86,87]
		Proteobacteria	*Escherichia coli* *Enterobacter cloacae* *Klebsiella pneumoniae*		
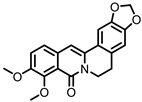 Oxyberberine	Berberine	Firmicutes	*Lactobacillus acidophilus* *Streptococcus aureus*	in vivo	[88]
		Actinobacteria	*Bifidobacterium longum*		
		Proteobacteria	*Escherichia coli* *Pseudomonas aeruginosa*		
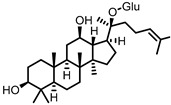 Compound K (CK)	Ginsenoside Rb1	Firmicutes	*Eubacterium*	in vitro, in vivo	[89,90,91,92,93,94]
		Actinobacteria	*Bifidobacterium*		
		Bacteroidetes	*Bacteroides*		
		Fusobacteria	*Fusobacterium*		

^a^ in vitro experiments are performed with berberine, as dihydroberberine is known to be re-oxidized to berberine after absorption.

## References

[1] Ng S.C., Shi H.Y., Hamidi N., Underwood F.E., Tang W., Benchimol E.I., Panaccione R., Ghosh S., Wu J.C.Y., Chan F.K.L. (2017). Worldwide incidence and prevalence of inflammatory bowel disease in the 21st century: A systematic review of population-based studies. Lancet.

[2] Guan Q. (2019). A Comprehensive Review and Update on the Pathogenesis of Inflammatory Bowel Disease. J. Immunol. Res..

[3] Ramos G.P., Papadakis K.A. (2019). Mechanisms of Disease: Inflammatory Bowel Diseases. Mayo Clin. Proc..

[4] Waljee A.K., Wiitala W.L., Govani S., Stidham R., Saini S., Hou J., Feagins L.A., Khan N., Good C.B., Vijan S. (2016). Corticosteroid Use and Complications in a US Inflammatory Bowel Disease Cohort. PLoS ONE.

[5] Chudy-Onwugaje K.O., Christian K.E., Farraye F.A., Cross R.K. (2019). A State-of-the-Art Review of New and Emerging Therapies for the Treatment of IBD. Inflamm. Bowel Dis..

[6] Hazel K., O’Connor A. (2020). Emerging treatments for inflammatory bowel disease. Ther. Adv. Chronic Dis..

[7] Caldeira L.D.F., Borba H.H., Tonin F.S., Wiens A., Fernandez-Llimos F., Pontarolo R. (2020). Fecal microbiota transplantation in inflammatory bowel disease patients: A systematic review and meta-analysis. PLoS ONE.

[8] Basso P.J., Câmara N.O.S., Sales-Campos H. (2019). Microbial-Based Therapies in the Treatment of Inflammatory Bowel Disease—An Overview of Human Studies. Front. Pharmacol..

[9] Tan P., Li X., Shen J., Feng Q. (2020). Fecal Microbiota Transplantation for the Treatment of Inflammatory Bowel Disease: An Update. Front. Pharmacol..

[10] Hou Q., Ye L., Liu H., Huang L., Yang Q., Turner J.R., Yu Q. (2018). Lactobacillus accelerates ISCs regeneration to protect the integrity of intestinal mucosa through activation of STAT3 signaling pathway induced by LPLs secretion of IL-22. Cell Death Differ..

[11] Wilck N., Matus M.G., Kearney S.M., Olesen S.W., Forslund K., Bartolomaeus H., Haase S., Mähler A., Balogh A., Markó L. (2017). Salt-responsive gut commensal modulates TH17 axis and disease. Nature.

[12] Zelante T., Iannitti R.G., Cunha C., De Luca A., Giovannini G., Pieraccini G., Zecchi R., D’Angelo C., Massi-Benedetti C., Fallarino F. (2013). Tryptophan catabolites from microbiota engage aryl hydrocarbon receptor and balance mucosal reactivity via interleukin-22. Immunity.

[13] Alexeev E.E., Lanis J.M., Kao D.J., Campbell E.L., Kelly C.J., Battista K.D., Gerich M.E., Jenkins B.R., Walk S.T., Kominsky D.J. (2018). Microbiota-Derived Indole Metabolites Promote Human and Murine Intestinal Homeostasis through Regulation of Interleukin-10 Receptor. Am. J. Pathol..

[14] Venkatesh M., Mukherjee S., Wang H., Li H., Sun K., Benechet A.P., Qiu Z., Maher L., Redinbo M.R., Phillips R.S. (2014). Symbiotic Bacterial Metabolites Regulate Gastrointestinal Barrier Function via the Xenobiotic Sensor PXR and Toll-like Receptor 4. Immunity.

[15] Dodd D., Spitzer M.H., Van Treuren W., Merrill B.D., Hryckowian A.J., Higginbottom S.K., Le A., Cowan T.M., Nolan G.P., Fischbach M.A. (2017). A gut bacterial pathway metabolizes aromatic amino acids into nine circulating metabolites. Nat. Cell Biol..

[16] Elsden S.R., Hilton M.G., Waller J.M. (1976). The end products of the metabolism of aromatic amino acids by clostridia. Arch. Microbiol..

[17] Wikoff W.R., Anfora A.T., Liu J., Schultz P.G., Lesley S.A., Peters E.C., Siuzdak G. (2009). Metabolomics analysis reveals large effects of gut microflora on mammalian blood metabolites. Proc. Natl. Acad. Sci. USA.

[18] Smith E.A., Macfarlane G.T. (1996). Enumeration of human colonic bacteria producing phenolic and indolic compounds: Effects of pH, carbohydrate availability and retention time on dissimilatory aromatic amino acid metabolism. J. Appl. Bacteriol..

[19] Aoki R., Aoki-Yoshida A., Suzuki C., Takayama Y. (2018). Indole-3-Pyruvic Acid, an Aryl Hydrocarbon Receptor Activator, Suppresses Experimental Colitis in Mice. J. Immunol..

[20] Wlodarska M., Luo C., Kolde R., D’Hennezel E., Annand J.W., Heim C.E., Krastel P., Schmitt E.K., Omar A.S., Creasey E.A. (2017). Indoleacrylic Acid Produced by Commensal Peptostreptococcus Species Suppresses Inflammation. Cell Host Microbe.

[21] Gaya P., Peirotén Á., Medina M., Álvarez I., Landete J.M. (2018). Bifidobacterium pseudocatenulatum INIA P815: The first bacterium able to produce urolithins A and B from ellagic acid. J. Funct. Foods.

[22] Larrosa M., González-Sarrías A., Yáñez-Gascón M.J., Selma M.V., Azorín-Ortuño M., Toti S., Tomás-Barberán F., Dolara P., Espín J.C. (2010). Anti-inflammatory properties of a pomegranate extract and its metabolite urolithin-A in a colitis rat model and the effect of colon inflammation on phenolic metabolism. J. Nutr. Biochem..

[23] Komatsu W., Kishi H., Yagasaki K., Ohhira S. (2018). Urolithin A attenuates pro-inflammatory mediator production by suppressing PI3-K/Akt/NF-κB and JNK/AP-1 signaling pathways in lipopolysaccharide-stimulated RAW264 macrophages: Possible involvement of NADPH oxidase-derived reactive oxygen species. Eur. J. Pharmacol..

[24] Piwowarski J.P., Kiss A.K., Granica S., Moeslinger T. (2015). Urolithins, gut microbiota-derived metabolites of ellagitannins, inhibit LPS-induced inflammation in RAW 264.7 murine macrophages. Mol. Nutr. Food Res..

[25] Bobowska A., Granica S., Filipek A., Melzig M.F., Moeslinger T., Zentek J., Kruk A., Piwowarski J.P. (2020). Comparative studies of urolithins and their phase II metabolites on macrophage and neutrophil functions. Eur. J. Nutr..

[26] Singh R., Chandrashekharappa S., Bodduluri S.R., Baby B.V., Hegde B., Kotla N.G., Hiwale A.A., Saiyed T., Patel P., Vijay-Kumar M. (2019). Enhancement of the gut barrier integrity by a microbial metabolite through the Nrf2 pathway. Nat. Commun..

[27] Selma M.V., Beltrán D., Luna M.C., Vaquero M.R., García-Villalba R., Mira A., Espín J.C., Tomás-Barberán F.A. (2017). Isolation of Human Intestinal Bacteria Capable of Producing the Bioactive Metabolite Isourolithin A from Ellagic Acid. Front. Microbiol..

[28] Beltrán D., Romo-Vaquero M., Espín J.C., Tomás-Barberán F.A., Selma M.V. (2018). Ellagibacter isourolithinifaciens gen. nov., sp. nov., a new member of the family Eggerthellaceae, isolated from human gut. Int. J. Syst. Evol. Microbiol..

[29] Selma M.V., Beltrán D., García-Villalba R., Espín J.C., Tomás-Barberán F.A. (2014). Description of urolithin production capacity from ellagic acid of two human intestinal Gordonibacter species. Food Funct..

[30] Selma M.V., Tomás-Barberán F.A., Beltrán D., García-Villalba R., Espín J.C. (2014). *Gordonibacter urolithinfaciens* sp. nov., a urolithin-producing bacterium isolated from the human gut. Int. J. Syst. Evol. Microbiol..

[31] Jin J.-S., Zhao Y.-F., Nakamura N., Akao T., Kakiuchi N., Min B.-S., Hattori M. (2007). Enantioselective Dehydroxylation of Enterodiol and Enterolactone Precursors by Human Intestinal Bacteria. Biol. Pharm. Bull..

[32] Peirotén Á., Gaya P., Álvarez I., Bravo D., Landete J.M. (2019). Influence of different lignan compounds on enterolignan production by Bifidobacterium and Lactobacillus strains. Int. J. Food Microbiol..

[33] Gaya P., Peirotén Á., Medina M., Landete J.M. (2017). Bifidobacterium adolescentis INIA P784: The first probiotic bacterium capable of producing enterodiol from lignan extracts. J. Funct. Foods.

[34] Bravo D., Peirotén Á., Álvarez I., Landete J.M. (2017). Phytoestrogen metabolism by lactic acid bacteria: Enterolignan production by Lactobacillus salivarius and Lactobacillus gasseri strains. J. Funct. Foods.

[35] Clavel T., Lippman R., Gavini F., Doré J., Blaut M. (2007). Clostridium saccharogumia sp. nov. and Lactonifactor longoviformis gen. nov., sp. nov., two novel human faecal bacteria involved in the conversion of the dietary phytoestrogen secoisolariciresinol diglucoside. Syst. Appl. Microbiol..

[36] Clavel T., Henderson G., Engst W., Dorã J., Blaut M., Doré J. (2006). Phylogeny of human intestinal bacteria that activate the dietary lignan secoisolariciresinol diglucoside. FEMS Microbiol. Ecol..

[37] Clavel T., Borrmann D., Braune A., Doré J., Blaut M. (2006). Occurrence and activity of human intestinal bacteria involved in the conversion of dietary lignans. Anaerobe.

[38] Wang L.-Q., Meselhy M.R., Li Y., Qin G.-W., Hattori M. (2000). Human Intestinal Bacteria Capable of Transforming Secoisolariciresinol Diglucoside to Mammalian Lignans, Enterodiol and Enterolactone. Chem. Pharm. Bull..

[39] Corsini E., Dell’Agli M., Facchi A., De Fabiani E., Lucchi L., Boraso M.S., Marinovich M., Galli C.L. (2010). Enterodiol and Enterolactone Modulate the Immune Response by Acting on Nuclear Factor-κB (NF-κB) Signaling. J. Agric. Food Chem..

[40] Almousa A.A., Meurens F., Krol E.S., Alcorn J. (2018). Linoorbitides and enterolactone mitigate inflammation-induced oxidative stress and loss of intestinal epithelial barrier integrity. Int. Immunopharmacol..

[41] Camuesco D., Comalada M., Rodriguez-Cabezas M.E., Nieto A., Lorente M.D., Concha A., Zarzuelo A., Gálvez J. (2004). The intestinal anti-inflammatory effect of quercitrin is associated with an inhibition in iNOS expression. Br. J. Pharmacol..

[42] Comalada M., Camuesco D., Sierra S., Ballester I., Xaus J., Gálvez J., Zarzuelo A. (2005). In vivoquercitrin anti-inflammatory effect involves release of quercetin, which inhibits inflammation through down-regulation of the NF-κB pathway. Eur. J. Immunol..

[43] Kim D.-H., Kim S.-Y., Park S.-Y., Han M.J. (1999). Metabolism of Quercitrin by Human Intestinal Bacteria and Its Relation to Some Biological Activities. Biol. Pharm. Bull..

[44] Park S.Y., Kim J.H., Kim D.H. (2005). Purification and characterization of quercitrin-hydrolyzing α-L-rhamnosidase from Fusobacterium K-60, a human intestinal bacterium. J. Microbiol. Biotechnol..

[45] Mascaraque C., Aranda C., Ocón B., Monte M.J., Suárez M.D., Zarzuelo A., Marín J.J.G., Martínez-Augustin O., de Medina F.S. (2014). Rutin has intestinal antiinflammatory effects in the CD4+ CD62L+ T cell transfer model of colitis. Pharmacol. Res..

[46] Kwon K.H., Murakami A., Tanaka T., Ohigashi H. (2005). Dietary rutin, but not its aglycone quercetin, ameliorates dextran sulfate sodium-induced experimental colitis in mice: Attenuation of pro-inflammatory gene expression. Biochem. Pharmacol..

[47] Cruz T., Gálvez J., Ocete M., Crespo M., de Medina F.-H.S., Zarzuelo A. (1998). Oral administration of rutoside can ameliorate inflammatory bowel disease in rats. Life Sci..

[48] Bokkenheuser V.D., Shackleton C.H., Winter J. (1987). Hydrolysis of dietary flavonoid glycosides by strains of intestinal Bacteroides from humans. Biochem. J..

[49] Seo-Hyeon B., Yang-Jin H., Juwon S., Sung-Woon H., Dong-Hyun K. (2015). Metabolism of Rutin and Poncirin by Human Intestinal Microbiota and Cloning of Their Metabolizing. J. Microbiol. Biotechnol..

[50] Shin N.R., Moon J.S., Shin S.-Y., Li L., Lee Y.B., Kim T.-J., Han N.S. (2016). Isolation and characterization of human intestinal Enterococcus avium EFEL009 converting rutin to quercetin. Lett. Appl. Microbiol..

[51] Beekwilder J., Marcozzi D., Vecchi S., De Vos R., Janssen P., Francke C., Vlieg J.V.H., Hall R.D. (2009). Characterization of Rhamnosidases from Lactobacillus plantarum and Lactobacillus acidophilus. Appl. Environ. Microbiol..

[52] Riva A., Kolimár D., Spittler A., Wisgrill L., Herbold C.W., Abrankó L., Berry D. (2020). Conversion of Rutin, a Prevalent Dietary Flavonol, by the Human Gut Microbiota. Front. Microbiol..

[53] Kim M., Kim N., Han J. (2014). Metabolism of Kaempferia parviflora Polymethoxyflavones by Human Intestinal Bacterium Bautia sp. MRG-PMF1. J. Agric. Food Chem..

[54] Corrêa T.A.F., Rogero M.M., Hassimotto N.M.A., Lajolo F.M. (2019). The Two-Way Polyphenols-Microbiota Interactions and Their Effects on Obesity and Related Metabolic Diseases. Front. Nutr..

[55] Braune A., Blaut M. (2016). Bacterial species involved in the conversion of dietary flavonoids in the human gut. Gut Microbes.

[56] Jang S.-E., Choi J.-R., Han M.J., Kim N.-H. (2016). The Preventive and Curative Effect of Cyanidin-3β-D-Glycoside and Its Metabolite Protocatechuic Acid Against TNBS-induced Colitis in Mice. Nat. Prod. Sci..

[57] Min S.-W., Ryu S.-N., Kim D.-H. (2010). Anti-inflammatory effects of black rice, cyanidin-3-O-β-d-glycoside, and its metabolites, cyanidin and protocatechuic acid. Int. Immunopharmacol..

[58] Ozdal T., Sela D.A., Xiao J., Boyacioglu D., Chen F., Capanoglu E. (2016). The Reciprocal Interactions between Polyphenols and Gut Microbiota and Effects on Bioaccessibility. Nutrients.

[59] Moco S., Martin F.-P.J., Rezzi S. (2012). Metabolomics View on Gut Microbiome Modulation by Polyphenol-rich Foods. J. Proteome Res..

[60] Rowland I., Gibson G., Heinken A., Scott K., Swann J., Thiele I., Tuohy K. (2018). Gut microbiota functions: Metabolism of nutrients and other food components. Eur. J. Nutr..

[61] Braune A., Blaut M. (2011). Deglycosylation of puerarin and other aromatic C-glucosides by a newly isolated human intestinal bacterium. Environ. Microbiol..

[62] Braune A., Blaut M. (2018). Catenibacillus scindens gen. nov., sp. nov., a C-deglycosylating human intestinal representative of the Lachnospiraceae. Int. J. Syst. Evol. Microbiol..

[63] Marín L., Miguélez E.M., Villar C.J., Lombó F. (2015). Bioavailability of Dietary Polyphenols and Gut Microbiota Metabolism: Antimicrobial Properties. BioMed Res. Int..

[64] Farombi E.O., Adedara I.A., Awoyemi O.V., Njoku C.R., Micah G.O., Esogwa C.U., Owumi S.E., Olopade J.O. (2016). Dietary protocatechuic acid ameliorates dextran sulphate sodium-induced ulcerative colitis and hepatotoxicity in rats. Food Funct..

[65] Crespo I., San-Miguel B., Mauriz J.L., de Urbina J.O., Almar M., Tuñón M.J., González-Gallego J. (2017). Protective Effect of Protocatechuic Acid on TNBS-Induced Colitis in Mice Is Associated with Modulation of the SphK/S1P Signaling Pathway. Nutrients.

[66] Hu R., He Z., Liu M., Tan J., Zhang H., Hou D.-X., He J., Wu S. (2020). Dietary protocatechuic acid ameliorates inflammation and up-regulates intestinal tight junction proteins by modulating gut microbiota in LPS-challenged piglets. J. Anim. Sci. Biotechnol..

[67] Larrosa M., Luceri C., Vivoli E., Pagliuca C., Lodovici M., Moneti G., Dolara P. (2009). Polyphenol metabolites from colonic microbiota exert anti-inflammatory activity on different inflammation models. Mol. Nutr. Food Res..

[68] Monagas M., Khan N., Andrés-Lacueva C., Urpí-Sardá M., Vázquez-Agell M., Lamuela-Raventós R.M., Estruch R. (2009). Dihydroxylated phenolic acids derived from microbial metabolism reduce lipopolysaccharide-stimulated cytokine secretion by human peripheral blood mononuclear cells. Br. J. Nutr..

[69] Miene C., Weise A., Glei M. (2011). Impact of Polyphenol Metabolites Produced by Colonic Microbiota on Expression of COX-2 and GSTT2 in Human Colon Cells (LT97). Nutr. Cancer.

[70] Pandurangan A.K., Mohebali N., Esa N.M., Looi C.Y., Ismail S., Saadatdoust Z. (2015). Gallic acid suppresses inflammation in dextran sodium sulfate-induced colitis in mice: Possible mechanisms. Int. Immunopharmacol..

[71] Pandurangan A.K., Mohebali N., Norhaizan M.E., Looi C.Y. (2015). Gallic acid attenuates dextran sulfate sodium-induced experimental colitis in BALB/c mice. Drug Des. Dev. Ther..

[72] Bayramoglu A., Kanbak G., Canbek M., Dokumac E. (2020). Gallic acid Reduces Experimental Colitis in Rats by Downregulation of Cathepsin and Oxidative Stress. Erciyes Med J..

[73] Khodayar B., Farzaei M.H., Hossein Abdolghaffari A., Bahramsoltani R., Baeeri M., Sabbagh Ziara-ni F., Mohammadi M., Rahimi R., Abdollahi M. (2018). The Protective Effect of the Gallic Acid Against TNBS-induced Ulcerative Colitis in Rats: Role of Inflammatory Parameters. J. Iran. Med. Counc..

[74] Zhu L., Gu P., Shen H. (2019). Gallic acid improved inflammation via NF-κB pathway in TNBS-induced ulcerative colitis. Int. Immunopharmacol..

[75] Marinov V.P., Tzaneva M.A., Zhelyazkova-Savova M.D., Gancheva S., Valcheva-Kuzmanova S.V. (2019). Effects of gallic acid in a rat model of inflammatory bowel disease induced by trinitrobenzenesulfonic acid. Bulg. Chem. Commun..

[76] Ávila M., Hidalgo M., Sánchez-Moreno C., Pelaez C., Requena T., de Pascual-Teresa S. (2009). Bioconversion of anthocyanin glycosides by Bifidobacteria and Lactobacillus. Food Res. Int..

[77] Hidalgo M., Oruna-Concha M.J., Kolida S., Walton G.E., Kallithraka S., Spencer J.P.E., Gibson G.R., De Pascual-Teresa S. (2012). Metabolism of Anthocyanins by Human Gut Microflora and Their Influence on Gut Bacterial Growth. J. Agric. Food Chem..

[78] Uhlenhut K., Högger P. (2012). Facilitated cellular uptake and suppression of inducible nitric oxide synthase by a metabolite of maritime pine bark extract (Pycnogenol). Free Radic. Biol. Med..

[79] Sun Y.N., Li W., Song S.B., Yan X.T., Zhao Y., Jo A.R., Kang J.S., Ho K.Y. (2016). A new phenolic derivative with soluble epoxide hydrolase and nuclear factor-kappaB inhibitory activity from the aqueous extract of Acacia catechu. Nat. Prod. Res..

[80] Kim H.S., Chung S., Song M.-Y., Lim C., Shin H., Hur J., Kwon H., Suh Y.-G., Kim E.-H., Shin D. (2020). Efficient and Divergent Enantioselective Syntheses of DHPVs and Anti-Inflammatory Effect on IEC-6 Cells. Molecules.

[81] Gu L., Li N., Li Q., Zhang Q., Wang C., Zhu W., Li J. (2009). The effect of berberine in vitro on tight junctions in human Caco-2 intestinal epithelial cells. Fitoterapia.

[82] Li N., Gu L., Qu L., Gong J., Li Q., Zhu W., Li J. (2010). Berberine attenuates pro-inflammatory cytokine-induced tight junction disruption in an in vitro model of intestinal epithelial cells. Eur. J. Pharm. Sci..

[83] Gu L., Li N., Gong J., Li Q., Zhu W., Li J. (2011). Berberine Ameliorates Intestinal Epithelial Tight-Junction Damage and Down-regulates Myosin Light Chain Kinase Pathways in a Mouse Model of Endotoxinemia. J. Infect. Dis..

[84] Takahara M., Takaki A., Hiraoka S., Adachi T., Shimomura Y., Matsushita H., Nguyen T.T.T., Koike K., Ikeda A., Takashima S. (2019). Berberine improved experimental chronic colitis by regulating interferon-γ- and IL-17A-producing lamina propria CD4+ T cells through AMPK activation. Sci. Rep..

[85] Li H., Feng C., Fan C., Yang Y., Yang X., Lu H., Lu Q., Zhu F., Xiang C., Zhang Z. (2020). Intervention of oncostatin M-driven mucosal inflammation by berberine exerts therapeutic property in chronic ulcerative colitis. Cell Death Dis..

[86] Jia L., Xue K., Liu J., Habotta O.A., Hu L., Moneim A.E.A., Caccamo D. (2020). Anticolitic Effect of Berberine in Rat Experimental Model: Impact of PGE2/p38 MAPK Pathways. Mediat. Inflamm..

[87] Feng R., Shou J.-W., Zhao Z.-X., He C.-Y., Ma C., Huang M., Fu J., Tan X.-S., Li X.-Y., Wen B.-Y. (2015). Transforming berberine into its intestine-absorbable form by the gut microbiota. Sci. Rep..

[88] Li C., Ai G., Wang Y., Lu Q., Luo C., Tan L., Lin G., Liu Y., Li Y., Zeng H. (2020). Oxyberberine, a novel gut microbiota-mediated metabolite of berberine, possesses superior anti-colitis effect: Impact on intestinal epithelial barrier, gut microbiota profile and TLR4-MyD88-NF-κB pathway. Pharmacol. Res..

[89] Kim D.-H. (2018). Gut microbiota-mediated pharmacokinetics of ginseng saponins. J. Ginseng Res..

[90] Yang L., Zou H., Gao Y., Luo J., Xie X., Meng W., Zhou H., Tan Z. (2020). Insights into gastrointestinal microbiota-generated ginsenoside metabolites and their bioactivities. Drug Metab. Rev..

[91] Wang C.-Z., Yao H., Zhang C.-F., Chen L., Wan J.-Y., Huang W.-H., Zeng J., Zhang Q.-H., Liu Z., Yuan J. (2018). American ginseng microbial metabolites attenuate DSS-induced colitis and abdominal pain. Int. Immunopharmacol..

[92] Joh E.-H., Lee I.-A., Jung I.-H., Kim D.-H. (2011). Ginsenoside Rb1 and its metabolite compound K inhibit IRAK-1 activation—The key step of inflammation. Biochem. Pharmacol..

[93] Li J., Zhong W., Wang W., Hu S., Yuan J., Zhang B., Hu T., Song G. (2014). Ginsenoside Metabolite Compound K Promotes Recovery of Dextran Sulfate Sodium-Induced Colitis and Inhibits Inflammatory Responses by Suppressing NF-κB Activation. PLoS ONE.

[94] Zhang J., Cao L., Wang H., Cheng X., Wang L., Zhu L., Yan T., Xie Y., Wu Y., Zhao M. (2015). Ginsenosides Regulate PXR/NF-κB Signaling and Attenuate Dextran Sulfate Sodium-Induced Colitis. Drug Metab. Dispos..

[95] Roager H.M., Licht T.R. (2018). Microbial tryptophan catabolites in health and disease. Nat. Commun..

[96] Monteleone I., Rizzo A., Sarra M., Sica G., Sileri P., Biancone L., Macdonald T.T., Pallone F., Monteleone G. (2011). Aryl Hydrocarbon Receptor-Induced Signals Up-regulate IL-22 Production and Inhibit Inflammation in the Gastrointestinal Tract. Gastroenterology.

[97] Wang Q., Yang K., Han B., Sheng B., Yin J., Pu A., Li L., Sun L., Yuan Q., Kunqiu Y. (2018). Aryl hydrocarbon receptor inhibits inflammation in DSS-induced colitis via the MK2/p-MK2/TTP pathway. Int. J. Mol. Med..

[98] Neavin D.R., Liu D., Ray B., Weinshilboum R.M. (2018). The Role of the Aryl Hydrocarbon Receptor (AHR) in Immune and Inflammatory Diseases. Int. J. Mol. Sci..

[99] Zenewicz L.A., Yancopoulos G.D., Valenzuela D.M., Murphy A., Stevens S., Flavell R.A. (2008). Innate and Adaptive Interleukin-22 Protects Mice from Inflammatory Bowel Disease. Immunity.

[100] Landete J. (2011). Ellagitannins, ellagic acid and their derived metabolites: A review about source, metabolism, functions and health. Food Res. Int..

[101] Candela M., Perna F., Carnevali P., Vitali B., Ciati R., Gionchetti P., Rizzello F., Campieri M., Brigidi P. (2008). Interaction of probiotic Lactobacillus and Bifidobacterium strains with human intestinal epithelial cells: Adhesion properties, competition against enteropathogens and modulation of IL-8 production. Int. J. Food Microbiol..

[102] Kawabata K., Yoshioka Y., Terao J. (2019). Role of Intestinal Microbiota in the Bioavailability and Physiological Functions of Dietary Polyphenols. Molecules.

[103] Vezza T., Rodríguez-Nogales A., Algieri F., Utrilla M.P., Rodriguez-Cabezas M.E., Galvez J. (2016). Flavonoids in Inflammatory Bowel Disease: A Review. Nutrients.

[104] Kaulmann A., Bohn T. (2016). Bioactivity of Polyphenols: Preventive and Adjuvant Strategies toward Reducing Inflammatory Bowel Diseases—Promises, Perspectives, and Pitfalls. Oxidative Med. Cell. Longev..

[105] Musumeci L., Maugeri A., Cirmi S., Lombardo G.E., Russo C., Gangemi S., Calapai G., Navarra M. (2019). Citrus fruits and their flavonoids in inflammatory bowel disease: An overview. Nat. Prod. Res..

[106] Ginwala R., Bhavsar R., Chigbu D.G.I., Jain P., Khan Z.K. (2019). Potential Role of Flavonoids in Treating Chronic Inflammatory Diseases with a Special Focus on the Anti-Inflammatory Activity of Apigenin. Antioxidants.

[107] Maleki S.J., Crespo J.F., Cabanillas B. (2019). Anti-inflammatory effects of flavonoids. Food Chem..

[108] Choy K.W., Murugan D., Leong X.-F., Abas R., Alias A., Mustafa M.R. (2019). Flavonoids as Natural Anti-Inflammatory Agents Targeting Nuclear Factor-Kappa B (NFκB) Signaling in Cardiovascular Diseases: A Mini Review. Front. Pharmacol..

[109] Sobhani M., Farzaei M.H., Kiani S., Khodarahmi R. (2020). Immunomodulatory; Anti-inflammatory/antioxidant Effects of Polyphenols: A Comparative Review on the Parental Compounds and Their Metabolites. Food Rev. Int..

[110] Ulusoy H.G., Sanlier N. (2020). A minireview of quercetin: From its metabolism to possible mechanisms of its biological activities. Crit. Rev. Food Sci. Nutr..

[111] Gan Y., Fu Y., Yang L., Chen J., Lei H., Liu Q. (2020). Cyanidin-3-O-Glucoside and Cyanidin Protect Against Intestinal Barrier Damage and 2,4,6-Trinitrobenzenesulfonic Acid-Induced Colitis. J. Med. Food.

[112] Talcott S., Talcott S., Sirven M. (2020). Moderate to Severe Ulcerative Colitis Results in Differential Metabolism of Cranberry Polyphenols by the Colon Microbiome Ex Vivo. Curr. Dev. Nutr..

[113] Kay C.D., Pereira-Caro G., Ludwig I.A., Clifford M.N., Crozier A. (2017). Anthocyanins and Flavanones Are More Bioavailable than Previously Perceived: A Review of Recent Evidence. Annu. Rev. Food Sci. Technol..

[114] Wiese S., Esatbeyoglu T., Winterhalter P., Kruse H.-P., Winkler S., Bub A., Kulling S.E. (2015). Comparative biokinetics and metabolism of pure monomeric, dimeric, and polymeric flavan-3-ols: A randomized cross-over study in humans. Mol. Nutr. Food Res..

[115] Appeldoorn M.M., Vincken J.-P., Aura A.-M., Hollman P.C.H., Gruppen H. (2009). Procyanidin Dimers Are Metabolized by Human Microbiota with 2-(3,4-Dihydroxyphenyl)acetic Acid and 5-(3,4-Dihydroxyphenyl)-γ-valerolactone as the Major Metabolites. J. Agric. Food Chem..

[116] Wang K., Feng X., Chai L., Cao S., Qiu F. (2017). The metabolism of berberine and its contribution to the pharmacological effects. Drug Metab. Rev..

[117] Kang Z., Zhonga Y., Wu T., Huang J., Zhao H., Liu D. (2021). Ginsenoside from ginseng: A promising treatment for inflammatory bowel disease. Pharmacol. Rep..

[118] Venegas D.P., De La Fuente M.K., Landskron G., González M.J., Quera R., Dijkstra G., Harmsen H.J.M., Faber K.N., Hermoso M.A. (2019). Short Chain Fatty Acids (SCFAs)-Mediated Gut Epithelial and Immune Regulation and Its Relevance for Inflammatory Bowel Diseases. Front. Immunol..

[119] Khan I., Ullah N., Zha L., Bai Y., Khan A., Zhao T., Che T., Zhang C. (2019). Alteration of Gut Microbiota in Inflammatory Bowel Disease (IBD): Cause or Consequence? IBD Treatment Targeting the Gut Microbiome. Pathogens.

